# The Anti-Cancer Effects of Mitochondrial-Targeted Triphenylphosphonium–Resveratrol Conjugate on Breast Cancer Cells

**DOI:** 10.3390/ph15101271

**Published:** 2022-10-15

**Authors:** Lingling Jiang, Han Yu, Chenwei Wang, Fujin He, Zhongqi Shi, Haohong Tu, Na Ning, Shaofeng Duan, Yunqi Zhao

**Affiliations:** 1 Wenzhou Municipal Key Lab of Applied Biomedical and Biopharmaceutical Informatics, Wenzhou-Kean University, Wenzhou 325060, China; 2 Zhejiang Bioinformatics International Science and Technology Cooperation Center, Wenzhou-Kean University, Wenzhou 325060, China; 3 School of Pharmacy, Henan University, Kaifeng 475004, China

**Keywords:** mitochondrial targeting, triphenylphosphonium, resveratrol, breast cancer, metabolomics

## Abstract

Breast cancer is the most commonly diagnosed cancer in women. Resveratrol, a naturally occurring phytochemical, shows great promise in developing novel anti-cancer therapies. This study hypothesized that the mitochondria-targeted delivery of resveratrol would increase its potency and induce mitochondria-mediated apoptosis. The targeted delivery of resveratrol was achieved by conjugating resveratrol to triphenylphosphonium (TPP). The anti-cancer effects of TPP-resveratrol were studied in the murine breast cancer 4T1 and the human breast cancer MDA-MB-231 cell lines. Flow cytometry was used to study apoptosis induction, cell cycle arrest, and mitochondrial membrane potential loss. The morphological changes in the mitochondria in MDA-MB-231 cells after TPP-resveratrol treatments were examined using transmission electron microscopy. Moreover, the changes in MDA-MB-231 cell metabolism after resveratrol and TPP-resveratrol treatments were studied using metabolomic analysis. We demonstrate that TPP-resveratrol significantly improved cytotoxicity in 4T1 cells and MDA-MB-231 cells by inducing apoptosis and mitochondrial membrane potential loss. Swollen and vacuolated mitochondria were observed after the TPP-resveratrol treatment. Meanwhile, TPP-resveratrol treatment down-regulated amino acid and energy metabolism and caused the dysfunction of purine and pyrimidine metabolism. Our results provide evidence supporting the targeted delivery of resveratrol to mitochondria and suggest that TPP-resveratrol may be an effective agent for breast cancer treatment.

## 1. Introduction

Breast cancer is one of the most life-threatening diseases with a high mortality rate among women [1]. Traditional breast cancer therapies, including surgery, chemotherapy, and radiotherapy, have been used to treat a variety of cancers and improve patients’ survival rates [2]. Although these treatments have been successful to some extent, they are not sufficient to eliminate all cancer cells and are associated with multiple adverse effects. Surgery, for example, does not prevent tumor recurrence and is usually followed by adjuvant therapy to reduce the risk of metastasis [3]. In addition, chemotherapy and radiotherapy treatments are often limited by cytotoxicity, multi-drug resistance, and poor selectivity [4]. Therefore, the development of novel, effective alternatives is imperative to reduce the side effects and improve the therapeutic efficacy of current breast cancer therapies.

In recent years, organelle-targeted drug delivery systems have attracted increasing attention as a means to reduce toxicity, lower drug dosages, improve therapeutic efficiency, and prevent drug resistance in cancer patients [5,6,7]. Mitochondria are crucial subcellular organelles that play an important role in cellular energy production, metabolism, and signaling. It has emerged as one of the most important targets for new drug design in cancer treatment [8,9]. Mitochondrial targeting can be classified into three types: lipophilic cation moieties, penetrating peptides, and mitochondrial targeting signal proteins [10]. One of the most widely used lipophilic cations is triphenylphosphonium (TPP). TPP conjugated with drugs, such as doxorubicin, coenzyme Q10, and curcumin, has been suggested as an alternative approach to cancer therapy [11,12,13]. 

Resveratrol, a natural polyphenolic phytoalexin, exhibits promising anti-cancer properties [14]. The anti-cancer effect may be due to apoptosis induction through the mitochondrial pathway. Resveratrol is also an inhibitor of mitochondrial complex III [9]. It could inhibit the activity of the adenine nucleotide transporter and decrease mitochondrial oxygen consumption [15]. Lucia Biasutto reported that TPP-resveratrol conjugates could significantly increase the cytotoxicity of colon cancer cells [16]. However, the specific mechanism of the TPP-resveratrol complex in cancer treatment remains poorly understood and needs further investigation.

Currently, metabolomics provides a promising analytical tool for identifying biomarkers and metabolic pathways altered in cancer and allows an in-depth investigation of the mechanisms associated with medical exposures [17]. It has been successfully applied to evaluate the effects of various drugs on breast cancer cells, lung adenocarcinoma cells, hepatocellular carcinoma cells, and prostate cancer cells [18,19,20,21]. At present, nuclear magnetic resonance (NMR), gas chromatography (GC), and liquid chromatography (LC) coupled with mass spectrometry (MS) are the most commonly used methods for metabolite detection [22]. In contrast to the other approaches, LC/MS offers higher efficiency in metabolic profiling, which is important for untargeted metabolomic research [19]. Therefore, in this study, we used LC/MS-based global metabolic profiling to investigate the effects of resveratrol and TPP-resveratrol on breast cancer cells.

## 2. Results

### 2.1. Cytotoxicity

To evaluate the anti-cancer effects of resveratrol and TPP-resveratrol, murine 4T1 and human MDA-MB-231 breast cancer cell line models were used. In the 4T1 cell cells, the IC_50_ values of resveratrol and TPP-resveratrol were 21.067 ± 3.7 and 16.216 ± 1.85 µM, respectively. The IC_50_s of resveratrol and TPP-resveratrol on the MDA-MB-231 cells were 29.97 ± 1.25 and 11.82 ± 1.46 µM, respectively. TPP did not show significant cytotoxicity to 4T1 and MDA-MB-231. Compared with resveratrol, the TPP-resveratrol conjugate significantly enhanced the anti-cancer effect of resveratrol (*p* < 0.05).

### 2.2. Mitochondrial Membrane Potential Loss

**Figure 1:**
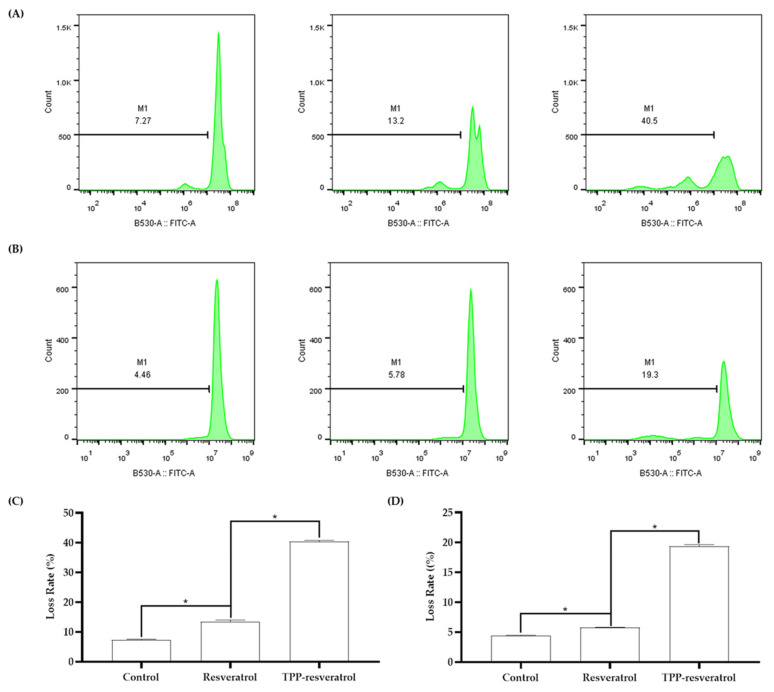
Representative flow cytometry histograms of 4T1 and MDA-MB-231 mitochondrial membrane potential loss after 50 μM resveratrol or TPP-resveratrol treatment. (**A**) The treatments induced membrane potential loss in 4T1 cells. (**B**) Membrane potential loss in MDA-MB-231 cells after the treatments. (**C**) Statistical analysis of the potential loss in 4T1 cells. (**D**) Statistical analysis of the potential loss in MDA-MB-231 cells (* *p* < 0.05).

Mitochondrial membrane potential loss was assessed by flow cytometry. Rhodamine 123 (Rh-123) could penetrate the cell membrane and accumulate in the mitochondria of living cells, emitting yellow-green fluorescence. In this study, 4T1 and MDA-MB-231 retained 92.73 ± 0.28% and 95.56 ± 0.05% fluorescence, respectively. When 4T1 and MDA-MB-231 cells were treated with 50 µM resveratrol for 6 h, the fluorescence decreased to 13.46 ± 0.55% and 5.78 ± 0.04%, respectively. After the TPP-resveratrol treatment (50 µM), the fluorescence was reduced to 40.33 ± 0.38% and 19.33 ± 0.25% in the 4T1 and MDA-MB-231 cell lines, respectively (Figure 1). 

### 2.3. Cell Apoptosis Induction

**Figure 2:**
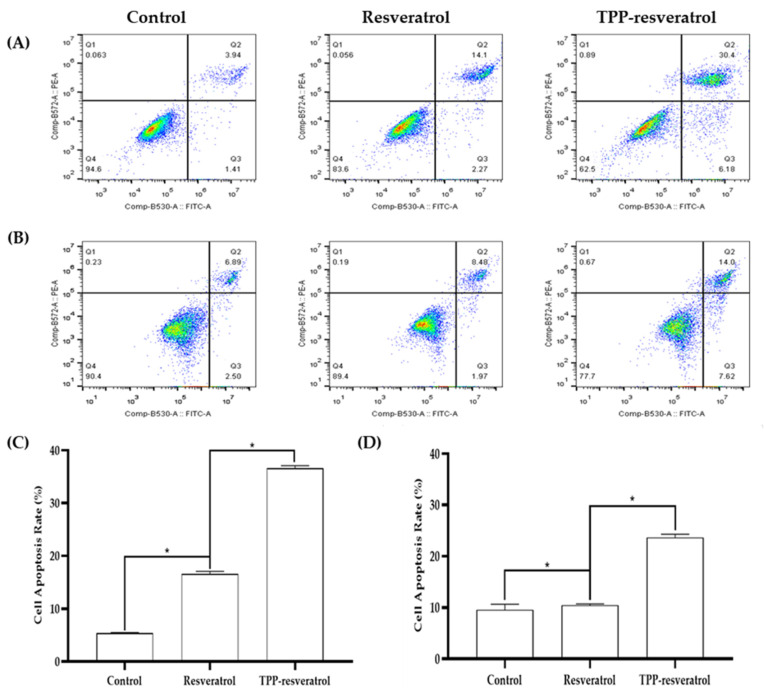
Representative results of apoptosis for resveratrol and TPP-resveratrol treatments of 4T1 and MDA-MB-231 breast cancer cells. Resveratrol and TPP-resveratrol treatments induced 4T1 (**A**) and MDA-MB-231 (**B**) cell apoptosis. Statistical analysis of apoptosis in 4T1 cancer cells (**C**) and MDA-MB-231 cancer cells (**D**) by flow cytometry (* *p* < 0.05).

Apoptosis induction was studied using an Annexin V-FITC Apoptosis Detection Kit via flow cytometry. Propidium iodide (PI) is a nucleic acid dye that can only penetrate the membrane of late apoptotic and dead cells. Annexin V and PI were combined to distinguish late apoptotic cells from dead cells [23]. After the 4T1 cells were treated with 50 µM resveratrol or TPP-resveratrol, the total apoptotic cells were 16.6 ± 0.47% and 36.6 ± 0.45%, respectively. In the MDA-MB-231 cell line, the apoptotic cell populations of resveratrol and TPP-resveratrol treatments were 10.4 ± 0.27% and 23.6 ± 0.62%, respectively (Figure 2). 

### 2.4. Cell Cycle Arrest

**Figure 3:**
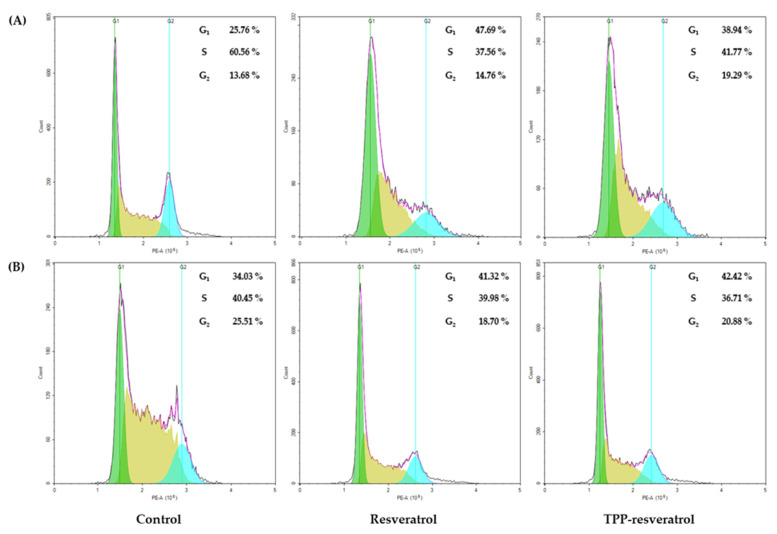
Representative results of cell cycle analysis for the resveratrol and TPP-resveratrol treatments of 4T1 and MDA-MB-231 breast cancer cells. (**A**) Both resveratrol and TPP-resveratrol induced 4T1 cell cycle arrest in the G_1_ phase. (**B**) The cell cycle was arrested in the G_1_ phase following resveratrol or TPP-resveratrol treatment in MDA-MB-231 breast cancer cells.

Cell cycle arrest was analyzed using flow cytometry to determine if resveratrol or TPP-resveratrol induced apoptosis-altered cell cycle distribution. The fluorescent dye PI could enter cells with damaged membranes and bind to their DNA. This strategy allowed the proportion of cells at each stage to be assessed according to their DNA content. In 4T1 cells, resveratrol arrested the cell cycle in the G_1_ phase (47.69%) and TPP-resveratrol arrested the cell cycle in the G_1_ (38.94%) and G_2_ (19.29%) phases. On the other hand, MDA-MB-231 cells were arrested in the G_1_ phase after resveratrol and TPP-resveratrol treatments (Figure 3). 

### 2.5. Mitochondrial Morphology

**Figure 4:**
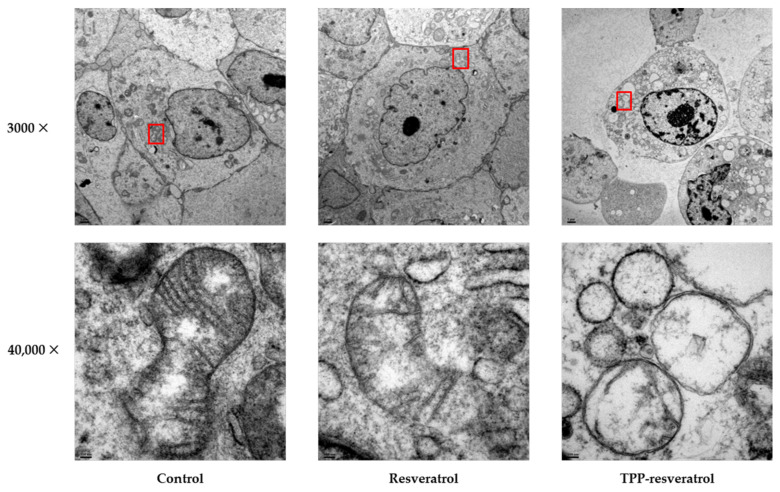
Transmission electron microscopy of MDA-MB-231 cancer cells treated with resveratrol or TPP-resveratrol at 50 μM. The selected higher-magnification regions are labeled in red squares.

Transmission electron microscopy (TEM) was used to evaluate the morphological changes in mitochondria after resveratrol and TPP-resveratrol treatments. In MDA-MB-231 cells, mitochondrial cristae were partly lost after resveratrol treatment. Furthermore, after TPP-resveratrol treatment, swollen and vacuolated mitochondria were observed (Figure 4). 

### 2.6. Metabolic Profiling Using Multivariate Analysis

**Figure 5:**
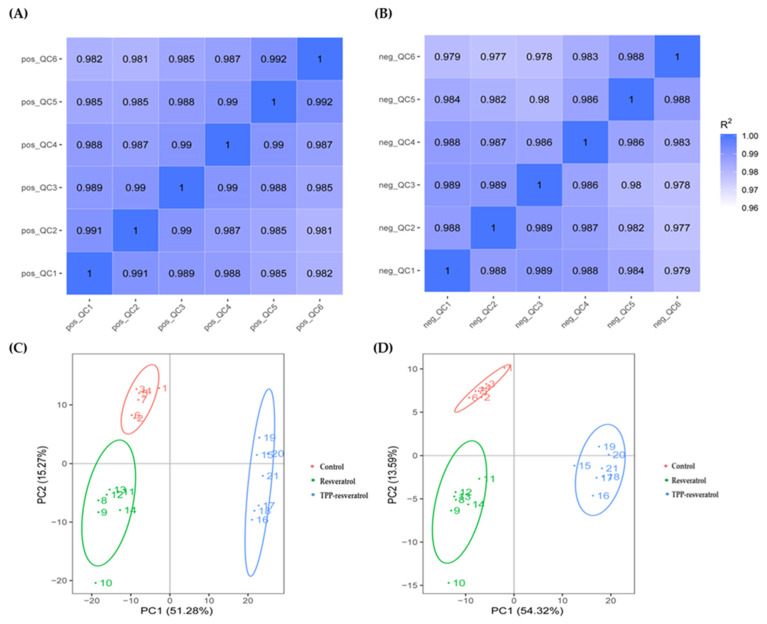
Pearson correlation coefficients of the quality control (QC) samples in the positive-ion mode (**A**) and the negative-ion mode (**B**). PCA score plots of the positive-ion mode (**C**) and the negative-ion mode (**D**).

The effects of resveratrol and the TPP-resveratrol conjugate on the human breast cancer MDA-MB-231 cells were analyzed using a metabolomics approach. The Pearson correlation coefficients between the quality control (QC) samples were calculated to assure data reliability [24]. As shown in Figure 5, the values were close to 1 in both the positive- and negative-ion modes, demonstrating that the obtained data were reliable. An unsupervised multivariate analysis method, principal component analysis (PCA), was carried out to obtain an overview of the metabolic pattern changes. As shown in Figure 5C,D, the metabolic patterns in the control, resveratrol, and TPP-resveratrol groups were primarily separated based on intracellular metabolites in both the positive and negative modes.

**Figure 6:**
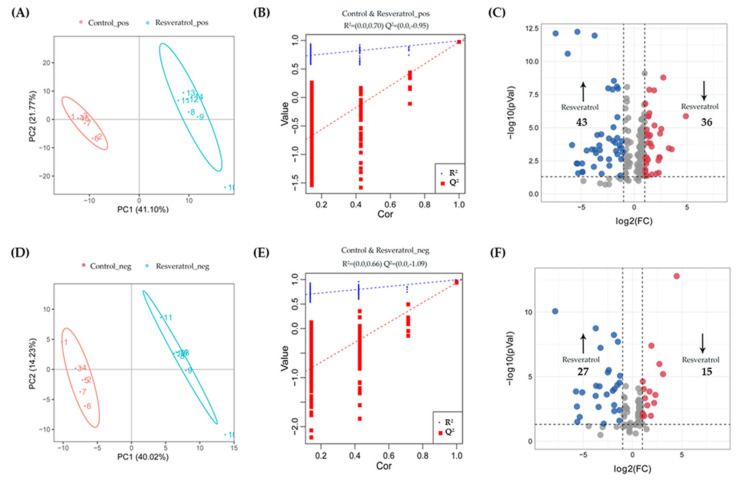
Differential metabolites selection in the MDA-MB-231 cells after resveratrol treatment. Score plots of OPLS-DA and permutation tests in the positive-ion mode (**A**,**B**) and negative-ion mode (**D**,**E**). The OPLS-DA models’ statistical parameters are as follows: (**A**) R2Y = 0.99, Q2Y = 0.98 and (**D**) R2Y = 0.99, Q2Y = 0.94. Volcano plots of resveratrol treatments compared with controls in the positive-ion mode (**C**) and negative-ion mode (**F**).

**Figure 7:**
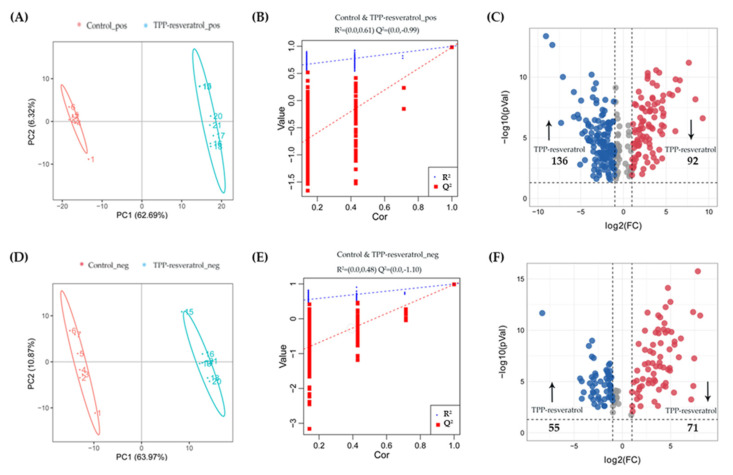
Differential metabolites selection in the MDA-MB-231 cells after TPP-resveratrol treatment. Score plots of OPLS-DA and permutation tests in the positive-ion mode (**A**,**B**) and negative-ion mode (**D**,**E**). The OPLS-DA models’ statistical parameters are as follows: (**A**) R2Y = 1 Q2Y = 0.98 and (**D**) R2Y = 1 Q2Y = 0.99. Volcano plots of TPP-resveratrol treatments compared with controls in the positive-ion mode (**C**) and negative-ion mode (**F**).

Orthogonal projections to latent structures–discriminant analysis (OPLS-DA) models were established to investigate the metabolite profiles in different groups (Figure 6 and Figure 7). Figure 6A,D and Figure 7A,D showed the score plots between the control/resveratrol groups and control/TPP-resveratrol groups in the positive- and negative-ion modes. The results indicated that the cancer cell metabolites were significantly altered after resveratrol and TPP-resveratrol treatments. Meanwhile, the permutation test was used to evaluate the OPLS-DA models’ fitting abilities and prediction abilities (Figure 6B,E and Figure 7B,E). The related parameters showed that R^2^ was higher than Q^2^, and the regression line of the Q^2^ points intersected the vertical at below zero, demonstrating that the models did not over-fit [19]. These results in our study followed the above standards, indicating that the OPLS-DA models were reliable.

### 2.7. Identification of Significantly Altered Metabolites

The criteria for identifying differential metabolites were as follows: VIP > 1, *p*-value < 0.05, and fold change >2 or <0.5. Among the differential metabolites in MDA-MB-231 cells, 70 for resveratrol and 191 for TPP-resveratrol treatment were significantly higher than those in the control group, while 51 for resveratrol and 163 for TPP-resveratrol treatment were significantly lower (Figure 6C,F and Figure 7C,F). This demonstrates that TPP-resveratrol treatment had a more pronounced effect on cellular metabolism than the resveratrol group.

**Figure 8:**
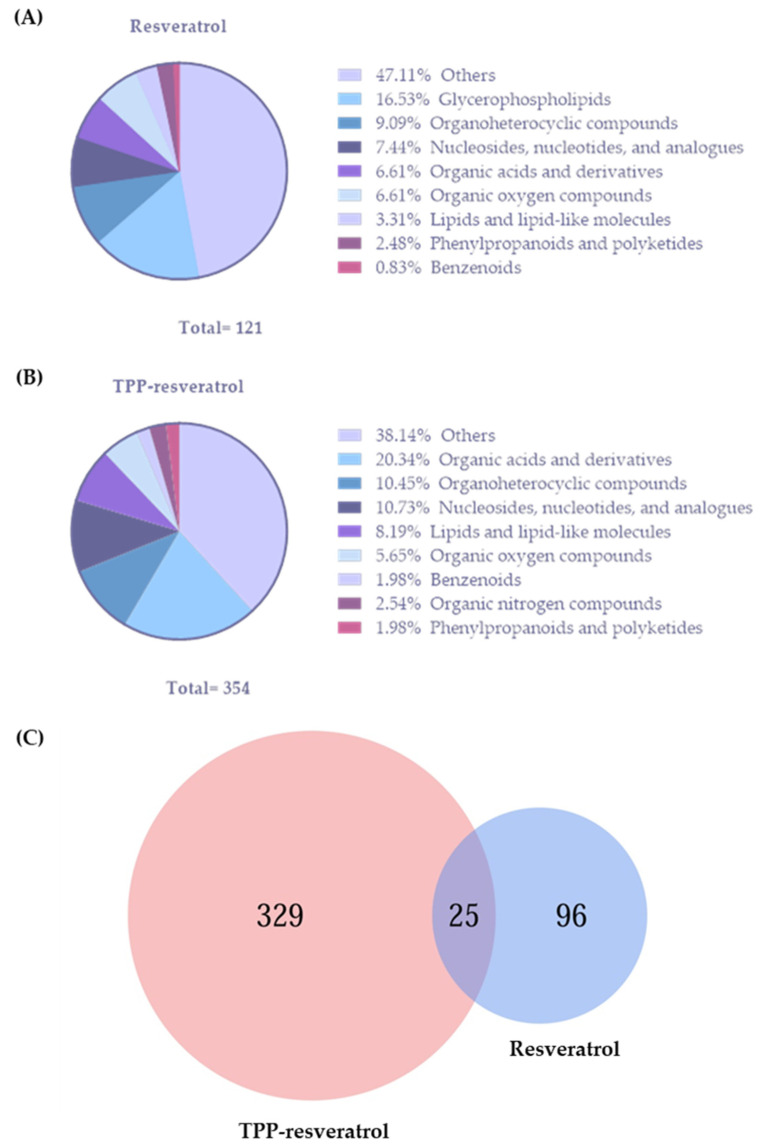
Classes of metabolites identified in the resveratrol group (**A**) and the TPP-resveratrol group (**B**). (**C**) Venn diagram showing the common and unique features between the resveratrol and TPP-resveratrol treatments.

The significantly altered metabolites included glycerophospholipids, organoheterocyclic compounds, nucleosides, nucleotides and analogs, organic acids and derivatives, organic oxygen compounds, lipids and lipid-like molecules, phenylpropanoids and polyketides, benzenoids, and organic nitrogen compounds (Figure 8A,B). In the Venn diagram analysis, 25 features were identified as common to both groups, while 329 and 96 were unique features to the TPP-resveratrol and resveratrol groups, respectively (Figure 8C). In addition, these differential metabolites were selected for further analysis.

The predominant compounds detected in the resveratrol and TPP-resveratrol groups were glycerophospholipids (16.53%, Figure 8A) and organic acids and derivatives (20.34%, Figure 8B), respectively. The up-regulated glycerophospholipids in the MDA-MB-231 cells are listed in Table 1. Table 2 shows biologically relevant metabolites and pathways that changed significantly in the cells after treatment with resveratrol and TPP-resveratrol. Among them, 11 metabolites belonging to organic oxygen compounds, organic acids and their derivatives, organoheterocyclic compounds, nucleosides, nucleotides and analogs, and organoheterocyclic compounds were increased. Thirty-eight metabolites associated with organic acids and derivatives, nucleosides, nucleotides and analogs, organic nitrogen compounds, and organoheterocyclic compounds were decreased compared with the control group.

### 2.8. Perturbed Metabolic Pathways Caused by Resveratrol and TPP-Resveratrol Exposure

**Figure 9:**
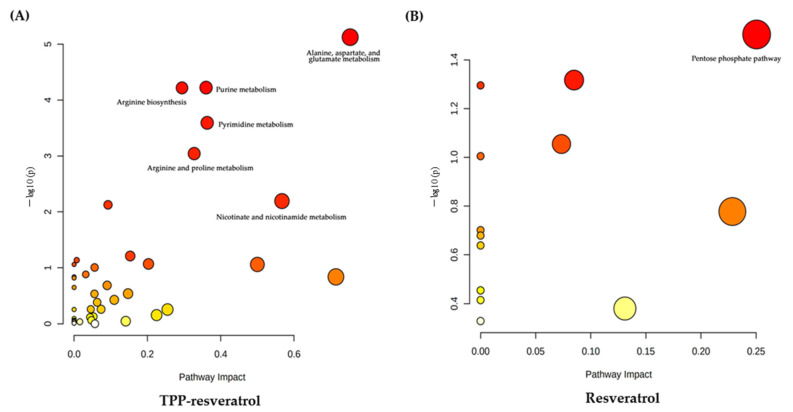
Summary of pathway analysis using MetaboAnalyst 5.0. (**A**) TPP-resveratrol treatment group. (**B**) Resveratrol treatment group.

A comprehensive metabolic network analysis was performed using MetabolAnalyst 5.0, revealing the most relevant pathways affected by resveratrol and TPP-resveratrol. Compared with the control group, alanine, aspartate and glutamate metabolism, purine metabolism, arginine biosynthesis, pyrimidine metabolism, arginine and proline metabolism, and nicotinate and nicotinamide metabolism were the significantly altered pathways in the TPP-resveratrol group (Figure 9A). In the resveratrol group, the impact value was low, even though the most significantly altered pathway was the pentose phosphate pathway (Figure 9B). Information regarding the relevant metabolites in these pathways and the tricarboxylic acid cycle (TCA cycle) is shown in Table 2.

## 3. Discussion

Previous studies have shown that resveratrol has an anti-cancer effect against breast cancer [25,26]. Our study formulated a mitochondria-targeted TPP-resveratrol conjugate and studied its efficacy on 4T1 and MDA-MB-231 cell lines. We showed that the conjugate could enhance the anti-cancer effect of resveratrol. In cytotoxicity assays, TPP-resveratrol reduced the IC_50_ of human MDA-MB-231 cells by nearly three-fold. The IC_50_ of murine 4T1 cells was also significantly reduced by the TPP-resveratrol formulation. These results suggest that the therapeutic effects of TPP-resveratrol at lower doses are comparable to those of resveratrol at higher levels.

Mitochondria, the sites for producing adenosine triphosphate (ATP), play important roles in regulating important signal transduction and metabolic processes associated with calcium homeostasis, reduction–oxidation (redox) potentials, and steroid hormone biosynthesis [27]. The mitochondrial dysfunction induced by growth factor changes, DNA damage, calcium overload, and cytoskeleton alterations would disrupt the mitochondrial homeostasis and induce type I programmed cell death (mitochondria-mediated apoptosis) [28,29,30]. Our results showed a high apoptotic rate by flow cytometry in these two cell lines after treatment with resveratrol or TPP-resveratrol. We also evaluated mitochondrial functioning via changes in the mitochondrial membrane potential. The results were consistent with those of apoptosis analysis. Moreover, we observed significant morphological changes in mitochondria after treatment with TPP-resveratrol. Therefore, TPP-resveratrol exhibited mitochondrial targeting and higher anti-cancer activity. Previous studies showed that resveratrol could arrest MDA-MB-231 cells in the G_1_ phase. These results are consistent with our data and indicate that the same signaling pathways were regulated [31,32].

Resveratrol has the ability to alter membrane lipids [33]. It has been reported that resveratrol-mediated lipidomic alterations can significantly affect glycerophospholipid metabolism [34]. Luciana Gomes found that phosphatidylcholine (PC) and phosphatidylethanolamine (PE) were decreased in MDA-MB-231 cells after resveratrol treatment [35]. Our study observed an increasing tendency in glycerophospholipids, including lysophosphatidylcholine (LPC) and lysophosphatidylethanolamine (LPE), as shown in Table 1. In apoptotic cells, the progressive degradation of PC is likely to result in LPC generation [36]. Interestingly, TPP-resveratrol exerted a greater impact on MDA-MB-231 cells’ metabolism than resveratrol, which had fundamentally different metabolic phenotypes. TPP, as a lipophilic cation, has been widely used in mitochondrial-targeting cancer therapeutics [37,38]. A recent study reported that resveratrol-encapsulated TPP liposomes enhanced the anti-cancer efficacy and mitochondrial depolarization in B16F10 cells [39]. However, the actual effect of TPP-resveratrol on the metabolism of breast cancer cells is still unclear. Cell metabolomics based on LC-MS/MS were used to investigate the metabolic mechanism of MDA-MB-231 cells after resveratrol or TPP-resveratrol treatment. The results indicated that TPP-resveratrol-induced apoptosis perturbed the amino acid and base metabolic networks the most.

In addition to providing substrates for the tricarboxylic acid (TCA) cycle, amino acids contribute to a broad array of processes essential for cell proliferation [40]. In the TPP-resveratrol treatment group, alterations in 7 metabolites involved in arginine biosynthesis and 10 metabolites involved in arginine and proline metabolism were observed. According to Table 2, the contents of the majority of metabolites in these pathways decreased, except for ornithine, which showed the opposite trend compared with the control group. Ornithine is a non-essential amino acid synthesized in the cytosol and transported into the mitochondria to perform its biological function [41]. It is produced by the enzymatic action of arginase on arginine and is a crucial substrate for proline synthesis [42]. It was revealed that TPP-resveratrol treatment decreased ornithine utilization and blocked the synthesis of arginine and proline. In terms of alanine, aspartate, and glutamate metabolism, the levels of 11 metabolites were decreased in the TPP-resveratrol group (Table 2). The biosynthesis of amino acids, such as aspartate and glutamate, is an important intermediate of the TCA cycle in the mitochondria [43]. The conjugation of a TPP group to various small molecules can facilitate mitochondrial targeting [44]. Our study also observed a significant perturbation of the TCA cycle in the TPP-resveratrol-treated cells. As shown in Table 2, the contents of TCA cycle-related metabolites (succinic acid, cis-aconitic acid, fumaric acid, citric acid, and malic acid) were decreased compared with those in the control group. Furthermore, a downward trend was observed in nicotinate and nicotinamide metabolism, which are also closely related to energy metabolism [45]. In this pathway, the levels of four metabolites, nicotinamide adenine dinucleotide (NAD), nicotinamide, 1-Methylnicotinamide, and nicotinamide adenine dinucleotide phosphate (NADP), decreased, as shown in Table 2, revealing that energy metabolism was down-regulated after TPP-resveratrol exposure.

Purine and pyrimidine metabolism are critical metabolic pathways in cell development, proliferation, and repair [46]. In this study, among the purine-related metabolites, the levels of 10 metabolites (guanosine 5′-diphosphate, l-glutamine, adenosine 3′5′-cyclic monophosphate, adenosine diphosphate, adenosine 5′-monophosphate, adenylosuccinate acid, inosine-5′-monophosphate, guanosine monophosphate, inosine, and adenine) were decreased while those of 5 metabolites (xanthine, deoxyinosine, guanine, deoxyguanosine, and guanosine) were increased (Table 2). In pyrimidine metabolism, the up-regulated metabolites included uridine monophosphate (UMP), cytidine-5′-monophosphate (CMP), and uracil, and the down-regulated metabolites included uridine diphosphate (UDP), L-glutamine, uridine, cytidine-5′-diphosphate (CDP), cytidine, dCDP, and dCMP. These results suggest that TPP-resveratrol exposure disrupted the homeostasis of purine and pyrimidine metabolism in breast cancer cells. The intermediate metabolites of these pathways are the crucial materials for the cell cycle, cell energy metabolism, and signal transduction [47,48,49]. Cancer cells with high proliferation and progression rates result in continuous high demand for energy and nutrients [50]. Therefore, our results demonstrate that TPP-resveratrol treatment might induce cell apoptosis by the down-regulation of amino acid and energy metabolism and the dysfunction of purine and pyrimidine metabolism.

## 4. Materials and Methods

### 4.1. Materials

RPMI-1640 medium and fetal bovine serum (FBS) were purchased from Corning (Shanghai, China). Penicillin–streptomycin was obtained from Gibco (Shanghai, China). Dojindo Molecular Technologies (Shanghai, China) provided the Cellstain^®^ Rhodamine 123 (Rh-123), Annexin V-FITC Apoptosis Detection, and PI/RNase Staining kits. Cell Counting Kit-8 (CCK-8) was purchased from GlpBio (Shanghai, China). Breast cancer cell lines 4T1 and MDA-MB-231 were commercially purchased from ProCell Life Science & Technology (CL-007) (Wuhan, Hubei, China) and the Peking Union Medical College Cell Culture Center (1101HUM-PUMC000014) (Beijing, China), respectively.

### 4.2. Synthesis of TPP-Resveratrol

**Scheme 1:**
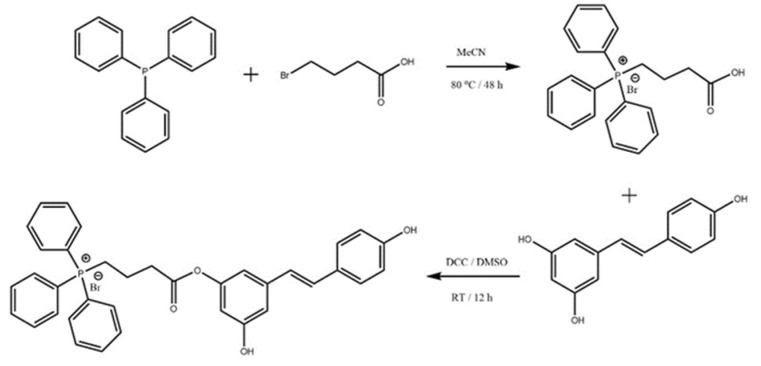
Synthesis of triphenylphosphonium–resveratrol conjugate.

The synthesis of the TPP-resveratrol conjugate is shown in Scheme 1. Triphenylphosphine (2.623 g, 10 mL, 1.0 eq), 4-bromobutyric acid (1.670 g, 10 mmol, 1.0 eq), and 40 mL of acetonitrile were added to a 100 mL round-bottomed flask. The reaction was then allowed to continue for 48 h at 80 °C. Upon completion, the reaction mixture was filtered to obtain a solid, which was washed with 40 mL × 3 filtrate and dried under a vacuum at room temperature overnight to produce the desired compound, TPP-COOH (2.446 g, 70% yield). ^1^H NMR (300 MHz, DMSO) δ 12.50 (s, 1H), 7.79 (s, 6H), 7.67 (d, *J* = 1.9 Hz, 1H), 7.54 (d, *J* = 8.7 Hz, 1H), 6.88 (d, *J* = 8.6 Hz, 1H), 6.40 (s, 1H), 6.18 (s, 1H), 5.58 (d, *J* = 7.7 Hz, 4H), 2.55 (s, 2H), 1.74 (s, 2H), and 1.24 (s, 2H).

To a 100 mL round-bottomed flask, TPP-COOH (0.856 g, 2 mmol, 1.0 eq), dicyclohexyl carbodiimide (0.619 g, 3 mmol, 1.5 eq), and 10 mL of dimethyl sulfoxide (DMSO) were added. Then, the reaction proceeded at room temperature for 2 h. The reaction liquid was filtered to remove the solid, and resveratrol (0.6847 g, 3 mmol, 1.5 eq) was added to the solution, followed by stirring at room temperature for 10 h. Upon completion, the reaction mixture was poured into 100 mL of ultrapure water to form a precipitate, which was filtered, washed with 40 mL of acetonitrile, and dried under a vacuum at room temperature overnight to produce the desired compound (0.9874 g, 77% yield). ^1^H NMR (300 MHz, DMSO) δ 9.21 (s, 1H), 8.25 (d, *J* = 7.7 Hz, 1H), 7.92–7.89 (m, 2H), 7.84–7.77 (m, 10H), 7.41 (s, 1H), 7.38 (s, 1H), 6.90 (s, 1H), 6.84 (s, 1H), 6.77 (s, 1H), 6.74 (s, 1H), 6.38 (d, *J* = 2.0 Hz, 2H), 6.11 (s, 1H), 5.59 (d, *J* = 8.0 Hz, 4H), 2.55 (s, 2H), 1.74 (s, 2H), and 1.24 (s, 2H).

### 4.3. Cytotoxicity

4T1 and MDA-MB-231 cells were cultured in RPMI-1640 medium containing 10% FBS and 1% penicillin–streptomycin. Both cell lines were maintained in a 37 °C incubator with 5% CO_2_. Cell Counting Kit-8 was used to test the cytotoxicity. 4T1 cells (2 × 10^3^ cells/well) and MDA-MB-231 cells (8 × 10^3^ cells/well) were seeded in 96-well plates overnight, followed by the addition of 10 μL of different concentrations of resveratrol (1, 5, 10, 50, 100, and 200 μM) and TPP-resveratrol (0.001, 0.01, 0.1, 1, 5, and 50 μM). After 48 h, 10 μL of CCK-8 solution was added to each well. Subsequently, the plate was incubated at 37 °C for 4 h. Finally, the optical density was measured at 450 nm and 650 nm using a microplate reader (VarioskanFlash-4.00.53, Thermo Scientific Inc., Shanghai, China).

### 4.4. Mitochondrial Membrane Potential Loss

A Cellstain^®^ Rhodamine 123 (Rh-123) kit was used to study mitochondrial functioning in the cells. 4T1 cells (1 × 10^6^ cells/well) and MDA-MB-231 cells (1 × 10^6^ cells/well) were seeded in 6-well plates overnight. After resveratrol and TPP-resveratrol (both 50 μM) treatment for 6 h at 37 °C, the cells were collected (>10^6^ cells) and washed. The cells were then resuspended in 1 mL of RPMI-1640 medium containing 20 μM of Rh-123 and incubated at 37 °C for 30 min. The cells were washed with phosphate buffer (PBS) once and analyzed by flow cytometry (NovoCyte 3000, Agilent; Software: Flowjo_V10, Novoexpress 1.5.0, Agilent, CA, USA).

### 4.5. Cell Apoptosis Induction

An Annexin V-FITC Apoptosis Detection Kit was used to examine the apoptotic induction of resveratrol and TPP-resveratrol (both 50 μM) in 4T1 and MDA-MB-231 cells. After 6 h of treatment, the cells collected by centrifugation (1000 rpm, 5 min) were washed in pre-cooled PBS and then suspended in 1 × Annexin V Binding Solution to prepare a cell suspension with a final concentration of 1 × 10^6^ cells/mL. Later, 5 μL of Annexin V-FITC conjugate was added to the cell suspension, followed by 5 μL of PI solution. The mixture was incubated at room temperature for 15 min in the dark. Finally, 400 μL of 1 × Annexin V Binding Solution was added, and the cells were detected by flow cytometry.

### 4.6. Cell Cycle Arrest

Cell cycle analysis was performed using a PI/RNase staining kit. The cells were treated with resveratrol or TPP-resveratrol (both 50 μM). After 6 h of treatment, the cells (>10^6^ cells/mL) were fixed with 70% ethanol at 4 °C for 2 h. Then, the cells were washed with 1 mL of PBS and centrifuged at 1000 rpm for 3 min. The supernatant was removed and 0.5 mL of working solution was added to the cell pellet. The mixture was then incubated for 30 min at 37 °C in the dark, followed by another 30 min at 4 °C. Finally, the cells were filtered through a nylon mesh to remove cell clumps from the sample. The distribution of cells in distinct cell cycle phases was determined by flow cytometry.

### 4.7. Transmission Electron Microscopy (TEM)

MDA-MB-231 cells were treated with resveratrol or TPP-resveratrol (both 50 μM) for 24 h and then collected in 2 mL micro-centrifuge tubes (>10^6^ cells). After centrifugation at 3000 rpm for 10 min, the supernatant was discarded and a pre-cooled 2.5% glutaraldehyde solution was slowly added. The samples were stored at 4 °C for 12 h. Then, the supernatant was discarded and the samples were washed with PBS and fixed with a 1% osmic acid solution for 1–2 h. Subsequently, the osmic acid waste solution was carefully removed. The samples were washed with PBS and dehydrated with an ethanol solution with gradient concentrations (including 30%, 50%, 70%, 80%, 90%, and 95%). Finally, the samples were treated with 100% ethanol for 20 min. Next, the samples were treated with pure acetone for 20 min, a mixed solution of embedding medium and acetone (*V*/*V* = 1/1) for 1 h, a mixed solution of embedding medium and acetone (*V*/*V* = 3/1) for 3 h, and pure embedding medium overnight. The embedded samples were obtained by heating the infiltrated samples at 70 °C overnight. The samples were sectioned using a LEICA EM UC7 ultramicrotome to obtain 70–90 nm sections. The sliced samples were then stained with lead citrate solution and 50% uranyl acetate-saturated ethanol for 10 min. After drying, TEM images were captured using a HITACHI H-7650 electron microscope to observe the mitochondrial morphology.

### 4.8. Intracellular Metabolite Extraction

The methanol–chloroform–water extraction method was used to extract intracellular metabolites [51]. For intracellular samples, the cells were rapidly washed three times with 10 mL of ice-cold PBS buffer. The cells were then trypsinized and centrifuged at 2000 rpm for 10 min at 4 °C. The supernatant was discarded, and 10 mL of PBS was used to wash the cell mass. The cell suspension was centrifuged at 2000 rpm for 5 min at 4 °C. The supernatant was discarded and the cell mass was bounced. Then, 450 uL of ice-cold methanol/chloroform (2:1, *v*/*v*) was added and sonicated on ice for 30 min. After that, 450 uL of ice-cold chloroform/water (1:1, *v*/*v*) was added, vortexed for 1 min, and incubated on ice for 15 min. Finally, the mixture was centrifuged at 12,000 rpm for 20 min at 4 °C. The supernatant was lyophilized and stored at −80 °C until it was analyzed. 

### 4.9. UHPLC-MS Analysis

Metabolite profiling was carried out using a Vanquish UHPLC system (ThermoFisher, Hannover, Germany) coupled with an Orbitrap Q Exactive TMHF-X mass spectrometer (ThermoFisher, Hannover, Germany). All chromatographic separations were performed using a Hypesil Gold Column (C18, 100 × 2.1 mm, 1.9 μm) with a flow rate of 0.2 mL/min. The mobile phase for the positive polarity mode consisted of 0.1% formic acid in water (A) and methanol (B). The negative polarity mode’s eluent A was 5 mM of ammonium acetate buffer (pH 9.0) and eluent B was methanol. The solvent gradient program was as follows: (i) 1.5 min with 2% B, (ii) 1.5 min with a linear gradient from 2% to 85% B, (iii) 7 min with a linear gradient from 85% to 100% B, and (iv) 2 min with 2% B. Separated metabolites were analyzed under both positive- and negative-ionization modes with a spray voltage of 3.5 kV, capillary temperature of 320 °C, sheath gas flow rate of 35 psi, aux gas flow rate of 10 L/min, S-lens RF level of 60, and aux gas heater temperature of 350 °C. 

### 4.10. Data Analysis and Metabolite Identification

Raw LC-MS/MS data were processed using a Compound Discoverer 3.1 (CD3.1, ThermoFisher) for peak alignment, peak picking, and quantitation of each metabolite. The main parameters were set as follows: retention time tolerance of 0.2 min, actual mass tolerance of 5 ppm, signal intensity tolerance of 30%, signal–noise ratio of 3, and minimum intensity of 100,000. After that, the peak intensities were normalized and the data were used to predict the molecular formula based on additive ions, molecular ion peaks, and fragment ions. Then, the peaks were matched with the mzCloud (https://www.mzcloud.org/, accessed on 10 June 2022), mzVault, and MassList databases to obtain accurate qualitative and quantitative results. A pooled quality control (QC) sample was prepared by mixing aliquots of all samples to assess the performance of the LC-MS instrument. The coefficient of variability (CV) was calculated, and compounds with CVs greater than 30% in the QC samples were removed [52].

Metabolites were annotated using the online Kyoto Encyclopedia of Genes and Genomes (KEGG), the Human Metabolome Database (HMDB), and the Lipid Metabolites and Pathways Strategy (LIPID Maps). Multivariate analysis, including principal components analysis (PCA) and orthogonal projection to latent structures–discriminant analysis (OPLS-DA), was conducted by metaX. Seven-fold cross-validation was applied to assess the performance of OPLS-DA. The fitness of model (R2) and predictive ability (Q2) values were calculated to monitor the models’ robustnesses. The values of the two parameters being close to 1.0 indicated an acceptable model. Important metabolites responsible for the class separation between the compared groups in the OPLS-DA scores plot were identified by the variable importance in the projection (VIP) scores. Volcano plots were used to filter metabolites of interest based on the fold changes (log2) and *p*-values (−log10). Metabolic pathway analysis was carried out by using Metaboanalyst 5.0 (https://www.metaboanalyst.ca/, accessed on 1 July 2022). 

## 5. Conclusions

In this study, a mitochondrial-targeted TPP-resveratrol was formulated. The results showed that TPP-resveratrol could trigger morphological changes in mitochondria and improve cytotoxicity by inducing apoptosis and mitochondrial membrane potential loss. Moreover, TPP-resveratrol could induce cell apoptosis by downregulating amino acid and energy metabolism, as well as by impairing purine and pyrimidine metabolism. Our results suggest that TPP-resveratrol may be an effective agent for breast cancer treatment.

## Figures and Tables

**Table 1:** Significantly altered glycerophospholipids in the MDA-MB-231 cells after resveratrol treatment.

Metabolites	m/z	RT ^1^ (min)	Formula	Trend
LPC ^2^ 12:0	440.276	7.246	C_20_H_42_NO_7_P	Up
LPC 14:0	512.300	8.299	C_22_H_46_NO_7_P	Up
LPC 15:0	482.324	8.738	C_23_H_48_NO_7_P	Up
LPC 16:0	540.331	9.187	C_24_H_50_NO_7_P	Up
LPC 16:1	538.316	8.550	C_24_H_48_NO_7_P	Up
LPC 17:1	552.331	8.971	C_25_H_50_NO_7_P	Up
LPC 18:0	568.362	10.058	C_26_H_54_NO_7_P	Up
LPC 18:1	566.347	9.174	C_26_H_52_NO_7_P	Up
LPC 18:2	564.331	8.844	C_26_H_50_NO_7_P	Up
LPC 20:3	590.347	9.152	C_28_H_52_NO_7_P	Up
LPC 20:5	586.316	8.285	C_28_H_48_NO_7_P	Up
LPC 22:6	612.332	8.709	C_30_H_50_NO_7_P	Up
LPE ^3^ 14:0	426.261	8.184	C_19_H_40_NO_7_P	Up
LPE 16:0	452.279	9.052	C_21_H_44_NO_7_P	Up
LPE 16:1	450.263	8.434	C_21_H_42_NO_7_P	Up
LPE 17:0	468.308	9.452	C_22_H_46_NO_7_P	Up
LPE 18:0	480.310	9.857	C_23_H_48_NO_7_P	Up
LPE 18:1	480.308	9.265	C_23_H_46_NO_7_P	Up
LPE 18:2	478.292	8.713	C_23_H_44_NO_7_P	Up
Lysopc 18:2	520.342	9.01	C_26_H_50_NO_7_P	Up

^1^ RT: retention time; ^2^ LPC: lysophosphatidylcholine; ^3^ LPE: lysophosphatidylethanolamine.

**Table 2:** Biologically relevant metabolites and pathways that changed significantly in the cells after resveratrol and TPP-resveratrol treatments.

Metabolic Pathways	Metabolites	Superclass	m/z	RT ^1^ (min)	Formula	Trend
Pentose phosphate pathway	d-Ribulose 5-phosphate	Organic oxygen compounds	229.012	1.207	C_5_H_11_O_8_P	Up
	6-Phospho-d-gluconate	Organic oxygen compounds	275.017	1.661	C_6_H_13_O_10_P	Up
TCA ^2^ cycle	Succinic acid	Organic acids and derivatives	117.019	2.402	C_4_H_6_O_4_	Down
	*cis*-Aconitic acid	Organic acids and derivatives	173.009	2.642	C_6_H_6_O_6_	Down
	Fumaric acid	Organic acids and derivatives	115.003	2.316	C_4_H_4_O_4_	Down
	Citric acid	Organic acids and derivatives	191.019	1.923	C_6_H_8_O_7_	Down
	Malic acid	Organic acids and derivatives	133.014	1.504	C_4_H_6_O_5_	Down
Alanine, aspartate, and glutamate metabolism	n-Acetyl-aspartic acid	Organic acids and derivatives	176.055	1.788	C_6_H_9_NO_5_	Down
	l-Aspartic acid	Organic acids and derivatives	134.045	1.313	C_4_H_7_NO_4_	Down
	Asparagine	Organic acids and derivatives	133.061	1.378	C_4_H_8_N_2_O_3_	Down
	l-Argininosuccinate	Organic acids and derivatives	289.115	1.336	C_10_H_18_N_4_O_6_	Down
	Adenylosuccinic acid	Nucleosides, nucleotides, and analogues	462.068	4.797	C_14_H_18_N_5_O_11_P	Down
	l-Glutamic acid	Organic acids and derivatives	148.060	1.312	C_5_H_9_NO_4_	Down
	γ- Aminobutyric acid (GABA)	Organic acids and derivatives	102.056	1.373	C_4_H_9_NO_2_	Down
	l-Glutamine	Organic acids and derivatives	147.076	1.378	C_5_H_10_N_2_O_3_	Down
Arginine biosynthesis	Ornithine	Organic acids and derivatives	133.097	1.139	C_5_H_12_N_2_O_2_	Up
	n-Acetylglutamic acid	Organic acids and derivatives	190.071	2.249	C_7_H_11_NO_5_	Down
Arginine and proline metabolism	Creatine	Organic acids and derivatives	132.077	1.363	C_4_H_9_N_3_O_2_	Down
	S-Adenosylmethionine	Nucleosides, nucleotides, and analogues	399.144	1.375	C_15_H_22_N_6_O_5_S	Down
	Spermidine	Organic nitrogen compounds	146.165	1.005	C_7_H_19_N_3_	Down
	Spermine	Organic nitrogen compounds	203.223	1.239	C_10_H_26_N_4_	Down
	d-Proline	Organic acids and derivatives	116.070	1.567	C_5_H_9_NO_2_	Down
	4-Guanidinobutanoic acid	Organic acids and derivatives	146.092	1.599	C_5_H_11_N_3_O_2_	Down
Purine metabolism	Guanosine 5′-diphosphate (GDP)	Nucleosides, nucleotides, and analogues	442.018	1.953	C_10_H_15_N_5_O_11_P_2_	Down
	Xanthine	Organoheterocyclic compounds	151.027	2.172	C_5_H_4_N_4_O_2_	Up
	Adenosine 3′5′-cyclic monophosphate	Nucleosides, nucleotides, and analogues	330.059	3.432	C_10_H_12_N_5_O_6_P	Down
	Adenosine diphosphate (ADP)	Nucleosides, nucleotides, and analogues	426.022	2.168	C_10_H_15_N_5_O_10_P_2_	Down
	Adenosine 5′-monophosphate (AMP)	Nucleosides, nucleotides, and analogues	346.056	1.596	C_10_H_14_N_5_O_7_P	Down
	Inosine-5′-monophosphate (IMP)	Nucleosides, nucleotides, and analogues	347.040	1.623	C_10_H_13_N_4_O_8_P	Down
	Deoxyinosine	Nucleosides, nucleotides, and analogues	253.092	3.674	C_10_H_12_N_4_O_4_	Up
	Guanosine monophosphate (GMP)	Organoheterocyclic compounds	362.051	1.572	C_10_H_14_N_5_O_8_P	Down
	Inosine	Nucleosides, nucleotides, and analogues	267.074	3.082	C_10_H_12_N_4_O_5_	Down
	Guanine	Organoheterocyclic compounds	152.057	4.753	C_5_H_5_N_5_O	Up
	Deoxyguanosine	Nucleosides, nucleotides, and analogues	266.090	3.665	C_10_H_13_N_5_O_4_	Up
	Guanosine	Nucleosides, nucleotides, and analogues	282.085	3.048	C_10_H_13_N_5_O_5_	Up
	Adenine	Organoheterocyclic compounds	136.062	2.729	C_5_H_5_N_5_	Down
Pyrimidine metabolism	Uridine diphosphate (UDP)	Nucleosides, nucleotides, and analogues	405.009	1.537	C_9_H_14_N_2_O_12_P_2_	Down
	Uridine monophosphate (UMP)	Nucleosides, nucleotides, and analogues	325.043	2	C_9_H_13_N_2_O_9_P	Up
	Uridine	Nucleosides, nucleotides, and analogues	243.062	2.171	C_9_H_12_N_2_O_6_	Down
	Cytidine-5′-diphosphate (CDP)	Nucleosides, nucleotides, and analogues	402.011	1.639	C_9_H_15_N_3_O_11_P_2_	Down
	Cytidine-5′-monophosphate (CMP)	Nucleosides, nucleotides, and analogues	322.045	1.653	C_9_H_14_N_3_O_8_P	Up
	Cytidine	Nucleosides, nucleotides, and analogues	242.080	1.369	C_9_H_13_N_3_O_5_	Down
	dCDP	Nucleosides, nucleotides, and analogues	386.017	1.731	C_9_H_15_N_3_O_10_P_2_	Down
	dCMP	Nucleosides, nucleotides, and analogues	308.063	1.386	C_9_H_14_N_3_O_7_P	Down
	Uracil	Organoheterocyclic compounds	111.020	1.764	C_4_H_4_N_2_O_2_	Up
Nicotinate and nicotinamide metabolism	Nicotinamide adenine dinucleotide (NAD)	Nucleosides, nucleotides, and analogues	662.102	1.917	C_21_H_27_N_7_O_14_P_2_	Down
	Nicotinamide	Organoheterocyclic compounds	123.055	1.587	C_6_H_6_N_2_O	Down
	1-Methylnicotinamide	Organoheterocyclic compounds	137.070	1.556	C_7_H_8_N_2_O	Down
	Nicotinamide adenine dinucleotide phosphate (NADP)	Nucleosides, nucleotides, and analogues	742.069	1.988	C_21_H_28_N_7_O_17_ P_3_	Down

^1^ RT: retention time; ^2^ TCA cycle: tricarboxylic acid cycle.

## Data Availability

Data are contained within the article.

## References

[1] Akram M., Iqbal M., Daniyal M., Khan A.U. (2017). Awareness and current knowledge of breast cancer. Biol. Res..

[2] Nounou M.I., ElAmrawy F., Ahmed N., Abdelraouf K., Goda S., Syed-Sha-Qhattal H. (2015). Breast Cancer: Conventional Diagnosis and Treatment Modalities and Recent Patents and Technologies. Breast Cancer Auckl..

[3] Dhankhar R., Vyas S.P., Jain A.K., Arora S., Rath G., Goyal A.K. (2010). Advances in novel drug delivery strategies for breast cancer therapy. Artif. Cells. Blood Substit. Immobil. Biotechnol..

[4] Chidambaram M., Manavalan R., Kathiresan K. (2011). Nanotherapeutics to overcome conventional cancer chemotherapy limitations. J. Pharm. Pharm. Sci..

[5] Liu C.G., Han Y.H., Kankala R.K., Wang S.B., Chen A.Z. (2020). Subcellular Performance of Nanoparticles in Cancer Therapy. Int. J. Nanomed..

[6] He Z., Zhang Y., Khan A.R., Ji J., Yu A., Zhai G. (2021). A novel progress of drug delivery system for organelle targeting in tumour cells. J. Drug Target..

[7] Gao P., Pan W., Li N., Tang B. (2019). Boosting Cancer Therapy with Organelle-Targeted Nanomaterials. ACS Appl. Mater. Interfaces.

[8] Xu Z., Chen X., Sun Z., Li C., Jiang B. (2019). Recent progress on mitochondrial targeted cancer therapy based on inorganic nanomaterials. Mater. Today Chem..

[9] Jeena M.T., Kim S., Jin S., Ryu J.H. (2019). Recent Progress in Mitochondria-Targeted Drug and Drug-Free Agents for Cancer Therapy. Cancers.

[10] Yu H., Ning N., Meng X., Chittasupho C., Jiang L., Zhao Y. (2022). Sequential Drug Delivery in Targeted Cancer Therapy. Pharmaceutics.

[11] Han M., Vakili M.R., Soleymani Abyaneh H., Molavi O., Lai R., Lavasanifar A. (2014). Mitochondrial delivery of doxorubicin via triphenylphosphine modification for overcoming drug resistance in MDA-MB-435/DOX cells. Mol. Pharm..

[12] Sena Ozbay H., Yabanoglu-Ciftci S., Baysal I., Gultekinoglu M., Can Eylem C., Ulubayram K., Nemutlu E., Topaloglu R., Ozaltin F. (2022). Mitochondria-targeted CoQ_10_ loaded PLGA-b-PEG-TPP nanoparticles: Their effects on mitochondrial functions of COQ8B^−/−^ HK-2 cells. Eur. J. Pharm. Biopharm..

[13] Reddy C.A., Somepalli V., Golakoti T., Kanugula A.K., Karnewar S., Rajendiran K., Vasagiri N., Prabhakar S., Kuppusamy P., Kotamraju S. (2014). Mitochondrial-targeted curcuminoids: A strategy to enhance bioavailability and anticancer efficacy of curcumin. PLoS ONE.

[14] Jiang Z., Chen K., Cheng L., Yan B., Qian W., Cao J., Li J., Wu E., Ma Q., Yang W. (2017). Resveratrol and cancer treatment: Updates. Ann. N. Y. Acad. Sci..

[15] Juan M.E., Wenzel U., Daniel H., Planas J.M. (2008). Resveratrol induces apoptosis through ROS-dependent mitochondria pathway in HT-29 human colorectal carcinoma cells. J. Agric. Food Chem..

[16] Biasutto L., Mattarei A., Marotta E., Bradaschia A., Sassi N., Garbisa S., Zoratti M., Paradisi C. (2008). Development of mitochondria-targeted derivatives of resveratrol. Bioorg. Med. Chem. Lett..

[17] Beger R.D. (2013). A review of applications of metabolomics in cancer. Metabolites.

[18] Dahabiyeh L.A., Mahmoud N.N., Al-Natour M.A., Safo L., Kim D.H., Khalil E.A., Abu-Dahab R. (2021). Phospholipid-Gold Nanorods Induce Energy Crisis in MCF-7 Cells: Cytotoxicity Evaluation Using LC-MS-Based Metabolomics Approach. Biomolecules.

[19] Hou L., Guan S., Jin Y., Sun W., Wang Q., Du Y., Zhang R. (2020). Cell metabolomics to study the cytotoxicity of carbon black nanoparticles on A549 cells using UHPLC-Q/TOF-MS and multivariate data analysis. Sci. Total Environ..

[20] Lindeque J.Z., Matthyser A., Mason S., Louw R., Taute C.J.F. (2018). Metabolomics reveals the depletion of intracellular metabolites in HepG2 cells after treatment with gold nanoparticles. Nanotoxicology.

[21] Sun H., Zhang A.H., Liu S.B., Qiu S., Li X.N., Zhang T.L., Liu L., Wang X.J. (2018). Cell metabolomics identify regulatory pathways and targets of magnoline against prostate cancer. J. Chromatogr. B Analyt. Technol. Biomed. Life Sci..

[22] Lee K.M., Jeon J.Y., Lee B.J., Lee H., Choi H.K. (2017). Application of Metabolomics to Quality Control of Natural Product Derived Medicines. Biomol. Ther..

[23] Crowley L.C., Marfell B.J., Scott A.P., Waterhouse N.J. (2016). Quantitation of Apoptosis and Necrosis by Annexin V Binding, Propidium Iodide Uptake, and Flow Cytometry. Cold Spring Harb. Protoc..

[24] Rao G., Sui J., Zhang J. (2016). Metabolomics reveals significant variations in metabolites and correlations regarding the maturation of walnuts (*Juglans regia* L.). Biol. Open.

[25] Wu H., Chen L., Zhu F., Han X., Sun L., Chen K. (2019). The Cytotoxicity Effect of Resveratrol: Cell Cycle Arrest and Induced Apoptosis of Breast Cancer 4T1 Cells. Toxins.

[26] Chin Y.T., Hsieh M.T., Yang S.H., Tsai P.W., Wang S.H., Wang C.C., Lee Y.S., Cheng G.Y., HuangFu W.C., London D. (2014). Anti-proliferative and gene expression actions of resveratrol in breast cancer cells in vitro. Oncotarget.

[27] Cho H., Cho Y.Y., Shim M.S., Lee J.Y., Lee H.S., Kang H.C. (2020). Mitochondria-targeted drug delivery in cancers. Biochim. Biophys. Acta Mol. Basis Dis..

[28] Abate M., Festa A., Falco M., Lombardi A., Luce A., Grimaldi A., Zappavigna S., Sperlongano P., Irace C., Caraglia M. (2020). Mitochondria as playmakers of apoptosis, autophagy and senescence. Semin. Cell Dev. Biol..

[29] Picca A., Calvani R., Coelho-Junior H.J., Marzetti E. (2021). Cell Death and Inflammation: The Role of Mitochondria in Health and Disease. Cells.

[30] Gibellini L., Moro L. (2021). Programmed Cell Death in Health and Disease. Cells.

[31] Yang M.F., Yao X., Chen L.M., Gu J.Y., Yang Z.H., Chen H.F., Zheng X., Zheng Z.T. (2020). Synthesis and biological evaluation of resveratrol derivatives with anti-breast cancer activity. Arch. Pharm..

[32] Bartolacci C., Andreani C., Amici A., Marchini C. (2018). Walking a Tightrope: A Perspective of Resveratrol Effects on Breast Cancer. Curr. Protein Pept. Sci..

[33] Brittes J., Lucio M., Nunes C., Lima J.L., Reis S. (2010). Effects of resveratrol on membrane biophysical properties: Relevance for its pharmacological effects. Chem. Phys. Lipids.

[34] Chen Z., Wang C., Lin C., Zhang L., Zheng H., Zhou Y., Li X., Li C., Zhang X., Yang X. (2021). Lipidomic Alterations and PPARalpha Activation Induced by Resveratrol Lead to Reduction in Lesion Size in Endometriosis Models. Oxid. Med. Cell Longev..

[35] Gomes L., Viana L., Silva J.L., Mermelstein C., Atella G., Fialho E. (2020). Resveratrol Modifies Lipid Composition of Two Cancer Cell Lines. Biomed Res. Int..

[36] Fuchs B., Schiller J., Cross M.A. (2007). Apoptosis-associated changes in the glycerophospholipid composition of hematopoietic progenitor cells monitored by 31P NMR spectroscopy and MALDI-TOF mass spectrometry. Chem. Phys. Lipids.

[37] Zielonka J., Joseph J., Sikora A., Hardy M., Ouari O., Vasquez-Vivar J., Cheng G., Lopez M., Kalyanaraman B. (2017). Mitochondria-Targeted Triphenylphosphonium-Based Compounds: Syntheses, Mechanisms of Action, and Therapeutic and Diagnostic Applications. Chem. Rev..

[38] Mallick A., More P., Ghosh S., Chippalkatti R., Chopade B.A., Lahiri M., Basu S. (2015). Dual drug conjugated nanoparticle for simultaneous targeting of mitochondria and nucleus in cancer cells. ACS Appl. Mater. Interfaces.

[39] Kang J.H., Ko Y.T. (2019). Enhanced Subcellular Trafficking of Resveratrol Using Mitochondriotropic Liposomes in Cancer Cells. Pharmaceutics.

[40] Lukey M.J., Katt W.P., Cerione R.A. (2017). Targeting amino acid metabolism for cancer therapy. Drug Discov. Today.

[41] Pita A.M., Fernandez-Bustos A., Rodes M., Arranz J.A., Fisac C., Virgili N., Soler J., Wakabayashi Y. (2004). Orotic aciduria and plasma urea cycle-related amino acid alterations in short bowel syndrome, evoked by an arginine-free diet. J. Parenter. Enter. Nutr..

[42] Sivashanmugam M., Jaidev J., Umashankar V., Sulochana K.N. (2017). Ornithine and its role in metabolic diseases: An appraisal. Biomed. Pharmacother..

[43] Engskog M.K., Ersson L., Haglof J., Arvidsson T., Pettersson C., Brittebo E. (2017). beta-N-Methylamino-L-alanine (BMAA) perturbs alanine, aspartate and glutamate metabolism pathways in human neuroblastoma cells as determined by metabolic profiling. Amino Acids.

[44] Biswas S., Dodwadkar N.S., Piroyan A., Torchilin V.P. (2012). Surface conjugation of triphenylphosphonium to target poly(amidoamine) dendrimers to mitochondria. Biomaterials.

[45] Xiao W., Wang R.S., Handy D.E., Loscalzo J. (2018). NAD(H) and NADP(H) Redox Couples and Cellular Energy Metabolism. Antioxid. Redox Signal..

[46] Lu Z., Li S., Aa N., Zhang Y., Zhang R., Xu C., Zhang S., Kong X., Wang G., Aa J. (2022). Quantitative analysis of 20 purine and pyrimidine metabolites by HILIC-MS/MS in the serum and hippocampus of depressed mice. J. Pharm. Biomed. Anal..

[47] Xu J., Chmela V., Green N.J., Russell D.A., Janicki M.J., Gora R.W., Szabla R., Bond A.D., Sutherland J.D. (2020). Selective prebiotic formation of RNA pyrimidine and DNA purine nucleosides. Nature.

[48] Oizel K., Tait-Mulder J., Fernandez-de-Cossio-Diaz J., Pietzke M., Brunton H., Lilla S., Dhayade S., Athineos D., Blanco G.R., Sumpton D. (2020). Formate induces a metabolic switch in nucleotide and energy metabolism. Cell Death Dis..

[49] Burnstock G. (2020). Introduction to Purinergic Signaling. Methods Mol. Biol..

[50] Leal-Esteban L.C., Fajas L. (2020). Cell cycle regulators in cancer cell metabolism. Biochim. Biophys. Acta Mol. Basis Dis..

[51] Cai A., Zheng H., Chen Z., Lin X., Li C., Yao Q., Bhutia Y.D., Ganapathy V., Chen R., Kou L. (2020). Synergism between SLC6A14 blockade and gemcitabine in pancreactic cancer: A ^1^H-NMR-based metabolomic study in pancreatic cancer cells. Biochem. J..

[52] Dai W., Xie D., Lu M., Li P., Lv H., Yang C., Peng Q., Zhu Y., Guo L., Zhang Y. (2017). Characterization of white tea metabolome: Comparison against green and black tea by a nontargeted metabolomics approach. Food Res. Int..

